# The impact of AR online shopping experience on customer purchase intention: An empirical study based on the TAM model

**DOI:** 10.1371/journal.pone.0309468

**Published:** 2024-08-26

**Authors:** Chunrong Guo, Xiaodong Zhang

**Affiliations:** 1 School of Economics and Management, Ningbo University of Technology, Ningbo, Zhejiang, China; 2 School of Economics and Management, Inner Mongolia Agricultural University, Hohhot, Inner Mongolia, China; Federal University of Goias: Universidade Federal de Goias, BRAZIL

## Abstract

Augmented Reality (AR) offers a rich business format, convenient applications, great industrial potential, and strong commercial benefits. The integration of AR technology with online shopping has brought tremendous changes to e-commerce. The Technology Acceptance Model (TAM) is a mature model for assessing consumer acceptance of new technologies, and applying it to evaluate the impact of AR online shopping experiences on consumer purchase intention is an urgently needed area of research. Firstly, the typical applications of AR in online shopping were reviewed, and the connotations and experiences of AR online shopping were summarized. Secondly, using the five types of AR online shopping experiences as antecedent variables, and perceived ease of use and perceived usefulness as intermediate variables, a theoretical model was constructed to explore the impact of AR online shopping experiences on customer purchase intentions, followed by an empirical study. Finally, suggestions were proposed for optimizing the online shopping experience to enhance purchase intentions. The article expands the application scenarios of the Technology Acceptance Model and enriches the theory of consumer behavior in Metaverse e-commerce.

## 1 Introduction

With the advent of the digital age, augmented reality (AR) technology has shown transformative potential across multiple industries, particularly in the realm of e-commerce [1]. Major retailers and brand corporations such as Google, Apple, Alibaba, Amazon, and Facebook have begun to employ AR technology to attract customers and boost sales. They actively integrate AR services into their business spheres to enhance customer awareness, brand engagement, and brand loyalty [2]. Surveys indicate that approximately 75% of consumers expect to experience AR services when shopping online, 71% state that they would shop more frequently if retailers utilized AR, and 40% are willing to pay more for products offered through AR. The AR market is projected to reach $50 billion by 2024 [3–6]. Due to their direct relationship with sales conversion rates and customer satisfaction, consumer shopping experiences have become one of the primary focuses of marketing management [7]. The AR strategy is crucial for merchants, especially in the highly competitive e-commerce market. A deep understanding and leveraging of AR technology’s potential can provide significant competitive advantages for businesses. Firstly, if studies find that AR experiences significantly enhance consumer purchase intentions, e-commerce platforms will be more inclined to invest in AR technology. Secondly, by identifying specific pain points in the user experience within AR applications through research, e-commerce businesses can optimize their AR applications, enhancing user satisfaction and loyalty. However, current research primarily explores the application of AR technology in e-commerce and its impact on consumer perceptions and behaviors [3–5, 8–10], aiming to understand the psychological and behavioral changes consumers undergo during AR experiences. Whang et al. (2021) adopted the concept of consumer control to investigate the mediating and moderating effects of AR experiences on purchase intention within the shopping environment for beauty products, with a focus on cognitive control and behavioral control [11]. However, comprehensive studies that specifically investigate the impact of AR online shopping experiences on consumer purchase intention and analyze the intrinsic mechanisms behind consumer acceptance of this new technology remain rare. Unlike previous studies, this research applies the Technology Acceptance Model (TAM) to explore how AR online shopping experiences affect consumer purchase intentions. It evaluates consumers’ attitudes towards AR online shopping experiences, how these experiences influence perceived usefulness and perceived ease of use, and how these factors translate into purchasing behavior. Although the TAM model has been widely used to assess consumer acceptance of new technologies, applying it to evaluate AR use in online shopping remains a largely unexplored area of research. This study introduces five types of AR online shopping experiences as antecedent variables to comprehensively analyze their impact on customer purchase intention. It aims to help e-commerce companies understand how different types of AR experiences influence consumer behavior, thereby enabling them to optimize user experiences in a targeted manner. The study explores the role of perceived ease of use and perceived usefulness as mediating variables between AR online shopping experiences and purchase intention, revealing the intrinsic mechanisms through which AR experiences affect consumer purchase intention and providing a theoretical basis for optimizing AR applications. Therefore, this study extends the application scenarios of the Technology Acceptance Model. Based on the empirical research results, specific optimization suggestions are proposed to enhance customer purchase intention. These suggestions offer actionable strategies for e-commerce marketers and service providers, improving the market competitiveness of e-commerce platforms. By analyzing AR online shopping experiences, this study enriches the theory of consumer behavior in Metaverse e-commerce and provides new perspectives and methods for future e-commerce research in the Metaverse environment.

AR (Augmented Reality) and VR (Virtual Reality) are key gateways into the metaverse, serving as the intersection and overlay of virtual and real worlds. These two technologies differ in their technical aspects, devices used, application fields, advantages, and potential, as shown in Table 1. AR, with its superior interactivity, real-time capabilities, visual effects, high portability, and ease of connectivity, demonstrates strong application value and development trends in areas such as e-commerce, shopping, marketing, advertising, social interaction, and entertainment.

**Table 1:** Comparison of AR and VR.

Comparison	Concept	Devices	Application Fields	Advantages
Augmented Reality(AR)	Overlaying virtual content onto the real world	Smartphones, tablets, monitors, AR glasses	Social, entertainment, shopping, advertising, industrial, navigation	Enhanced interactivity and real-time capabilities: Overlaying virtual content onto the real world allows users to interact with the virtual content and receive information and feedback in real time. Strong visualization: Transforms abstract data into visual formats, making it easier for users to understand and analyze. High portability: Features increased portability and usability.
Virtual Reality(VR)	Simulating virtual environments to create immersive experiences	Head-mounted displays	Gaming, education, healthcare, etc.	Strong sense of immersion: Allows users to fully immerse themselves in virtual environments, enhancing their engagement and entertainment. High fidelity: Deeply simulates real-world environments, enabling users to learn and practice in a safe, low-cost setting. Strong creativity: Creates virtual art and cultural experiences, allowing appreciation of art and culture from anywhere at any time.

## 2 Basic theories

### 2.1 AR online shopping

Augmented Reality technology originated in the 1990s, but it was not until the early 21st century, with the widespread adoption of smartphones and high-speed internet, that this technology began to be applied in the online shopping sector. Retailers and tech companies invested in image recognition improvements and 3D modeling technologies to enable a more realistic product experience for consumers. One of the earliest applications was a virtual fitting room that allowed users to try on clothes via a web camera. In 2017, IKEA launched an AR app named “IKEA Place” that allowed users to virtually place furniture in their homes to see how it would look in a real environment. After 2020, the use of AR technology in online shopping became more widespread. Besides virtual try-ons, it was also used for home decor, cosmetics selection, and even in some high-tech stores for AR virtual shopping assistants (see Table 2). E-commerce giants like Amazon and Alibaba integrated AR technology into their shopping platforms, providing a richer and more interactive online shopping experience. For instance, Amazon’s AR View feature allows users to virtually view products in their own living spaces. In the future, augmented reality will further merge with technologies such as virtual reality and blockchain to create a comprehensive metaverse digital shopping environment, offering users a fully immersive and interactive shopping experience.

**Table 2:** Applications and typical cases of augmented reality in online shopping.

Application Type	Product Type	Typical Cases	Application Effect
Virtual Try-On	Apparel and Accessories	Nike’s “Nike Fit”	Uses AR technology to help users measure their foot size using their smartphone camera and see how selected shoe styles look on their feet. The effectiveness and acceptance of this application depend on its realism, accuracy, and ease of use. Technological innovation positively impacts enhancing the online shopping experience and boosting consumer confidence.
Online Makeup Trial	Cosmetics	L’Oréal’s “L’Oréal Paris Virtual Try-On” or “ModiFace”	Allows users to see the effects of different colors and products on their own faces. The effectiveness and acceptance of this application depend on the realistic experience, convenience, and a wide range of product choices. AR applications greatly enhance the online shopping experience, receiving positive market feedback.
Furniture and Home Decor	Furniture and Decorative Items	IKEA’s “IKEA Place”	Allows users to view the placement of furniture in their homes in 3D. The effectiveness and acceptance mainly depend on the technical precision, user interface and ease of use, device compatibility, the extensiveness and diversity of the product library, network connection and performance requirements, marketing and customer education, customer feedback and ongoing improvements. The integration of real and virtual effects significantly enhances the shopping experience, with positive outcomes in practical application [12].
Car Display and Customization	Automobiles	Audi’s “Audi AR Visualizer” and other car manufacturers use AR technology to allow users to view different car models and customization options on their smartphones or tablets	Allows users to view different Audi models and explore a variety of customization options, such as body color and wheel design, through their smartphones or tablets. The effectiveness and acceptance of this feature primarily depend on the accuracy and practicality of the technology, the intuitiveness and ease of use of the user interface, device compatibility, the diversity of customization options, real-time interaction, marketing and user education, network performance and application stability, and the collection of feedback and improvements to the application.
Interactive Marketing and Advertising	Multiple Products	Sprite’s AR Interactive Ad Campaign “Let’s Be Clear”	Users experience unique augmented reality content through their smartphones. The effectiveness and acceptance mainly depend on the quality of the technology, creative content, user interface and ease of use, marketing strategies, target audience, compatibility and accessibility, value-added experience, feedback, and improvements. Sprite’s AR interaction is primarily targeted at young adults, especially Generation Z. The campaign focuses on transparency, individuality, and authenticity, encouraging consumers to enjoy Sprite at pivotal moments in their daily lives.

Despite the rapid development of AR online shopping in practice, there is currently no unified definition. Summarizing existing theoretical research and practical developments, AR online shopping is an innovative shopping method that combines the convenience of online shopping with the experiential aspect of physical shopping. It overlays virtual information and images in the consumer’s actual environment and displays them in 3D. This allows consumers to perceive and interact with virtual elements in a more realistic and three-dimensional way in real-time, providing a richer and more immersive shopping experience. Consequently, consumers can more accurately understand the appearance and functions of products before purchasing, thereby enhancing shopping efficiency and satisfaction [13, 14]. AR online shopping displays product elements in three-dimensional (3D) form and assimilates virtual objects into the physical reality, allowing users to experience the coexistence of real and virtual elements in the same space and interact with products in an enhanced manner [15, 16]. AR interaction technology enables users to virtually try, verify, and inspect products from various angles and size [17], It responds instantly to user actions such as rotating, zooming, or altering products, and any changes made during user interactions are immediately reflected in the AR interface. This instant interaction enhances the dynamism and enjoyment of shopping [18, 19], increases consumer engagement, and promotes sales in a heuristic and effective manner [20]. AR technology allows users to make personalized adjustments and trials according to their preferences and needs, turning customers into co-designers of the products they wish to purchase, thereby creating personalized products or customizing them in a personalized way [21].

Immersion refers to the degree to which an individual’s senses are cut off from the real world and replaced by a virtual simulation [16]. Initially a way for gamers to interact with their physical environment, immersive AR technology is now used to enhance e-commerce platforms through richer media experiences, simpler navigation, and the multidimensional and multisensory presentation of products [8], placing customers in a new immersive space. This allows users to navigate spatial locations via web browsers in an interactive simulated manner, experiencing the sensation of shopping in person at actual locations, thus creating a retail store shopping feel accessible from anywhere. This can evoke emotional, cognitive, and behavioral responses [9], enhancing enjoyment, perceived usefulness, and purchase intent. With rapid advancements in augmented reality technology, along with the swift development of VR, 5G/6G, Enhanced Mobile Broadband (eMBB), Ultra-Reliable Low-Latency Communications (URLLC), Artificial Intelligence (AI), and blockchain technology, immersive 3D experiences and multisensory communications blur the lines between virtual and physical worlds to form a metaverse of mixed reality [22]. Within the context of the metaverse, AR, VR, and XR (Extended Reality) have all made significant progress, considered the next generation of the internet or social media, poised to revolutionize shopping and marketing [23].

### 2.2 Experiential marketing

Intense competition has made the functional attributes of products and services increasingly similar, making experience a key differentiator among businesses, especially in the retail environment. A core goal for businesses is to create outstanding customer experiences. Experiential marketing has become a standard practice for many merchants [24]. It goes beyond the transactional level of traditional marketing of products and services, focusing instead on creating emotional and sensory connections with consumers. The core of experiential marketing is to create a comprehensive consumption experience. Experiential marketing includes five main dimensions. Sensory Experience (SENSE): Sensory experience focuses on stimulating the consumer’s senses—sight, hearing, smell, touch, and taste. By creating an appealing visual environment, playing pleasing music, offering unique tastes and scents, or providing tactile experiences, businesses can enhance consumers’ product perceptions and memories. Emotional Experience (FEEL): Emotional experience aims to evoke consumers’ emotional responses and emotional connections. For example, businesses can touch consumers’ hearts through emotionally resonant advertising, storytelling, or user experiences. This type of experience might be based on joy, surprise, nostalgia, or other emotions, with the goal of establishing a deeper emotional connection with consumers. Creative Cognitive Experience (THINK): The creative cognitive experience encourages consumers to actively think, explore, and innovate. This type of experience often stimulates consumers’ curiosity and imagination by solving problems, offering novel perspectives, or introducing unfamiliar concepts. For example, consumers’ thinking and engagement may be stimulated through interactive exhibitions, educational workshops, or innovative product demonstrations. Physical Experience, Actions, and Lifestyle (ACT): Physical experience involves consumer behaviors and direct interactions with products or services, including using products, participating in activities, or adopting specific lifestyles. For example, experiential retail stores or interactive exhibitions encourage consumers to engage with and experience the brand’s lifestyle. Social Identity Experience (RELATE): The social identity experience emphasizes the relationships between consumers and others, and how they define their social identities through brands. This can be achieved through interactions on social media, community events, or associations with certain cultures or groups. For example, some brands incorporate specific cultural values or social movements, making consumers feel like part of a larger group [25, 26]. In summary, experiential marketing creates a comprehensive and immersive consumer experience through these five dimensions, aiming to establish a deeper emotional connection between the brand and consumers [27].

In the field of online shopping, creating a unique online shopping experience has become key to attracting customers and maintaining customer loyalty. Experiential marketing in online shopping has now become a focus of attention for both academic researchers and practitioners. Key factors in building positive online experiences include vividness, interactivity, and uniqueness. However, achieving these objectives faces several challenges. On one hand, due to the complex cognitive structures of consumers, exploring the mechanisms behind consumer online buying behaviors is difficult. On the other hand, virtual experiences have certain limitations that directly impact customer purchasing behavior. Marketers should seek innovative methods to overcome these challenges, including the use of metaverse technologies such as augmented reality and virtual reality, enabling consumers to interact with virtual content in the real world and experience it in a holistic manner.

### 2.3 Technology Acceptance Model (TAM)

The Technology Acceptance Model (TAM), proposed by Davis in 1989 [28], has been a key theoretical framework widely used in the field of information systems since the late 1980s [29]. TAM aims to explain and predict user behavior in accepting and using new technologies [30]. The model suggests that an individual’s intention to use a technology is primarily determined by two main factors: Perceived Ease of Use (PEOU) and Perceived Usefulness (PU). Perceived Usefulness refers to the user’s belief that using a particular technology will enhance their job performance, meaning that a practical technology is more likely to be accepted and used by users [31]. Perceived Ease of Use refers to the user’s perception of how easy or difficult a technology is to use; if users believe a technology is easy to use, they are more likely to adopt it [32]. Perceived Usefulness is influenced by Perceived Ease of Use because, all other conditions being equal, a technology that is easier to use is more likely to be accepted.

Since its inception in 1991, the Technology Acceptance Model has generated over 1,000 related publications in the field of management, making it one of the most popular theoretical models [7]. TAM has also become an appropriate hypothesis model for studying the acceptance of AI technology in e-commerce [33]. Magsamen-Conrad et al. [34] have used Perceived Ease of Use to define the comfort level when using social networking platforms. Jacob and Pattusamy [35] have described how Perceived Usefulness indicates the extent to which using social networks can aid in sustaining people’s learning activities. If users believe that the technology behind an online shopping experience is useful and easy to use, they are more inclined to use that technology. However, the TAM model focuses only on the extrinsic motivations for technology use [23]. To enhance the explanatory power of TAM, researchers have expanded it by incorporating various external variables when using the model. This expansion allows studies to address the intrinsic motivations of users for using a particular technology. One such variable is Perceived Enjoyment, meaning if consumers enjoy the online shopping experience, they are likely to have a positive attitude towards the specific technology [36]. Another expansion of TAM involves the introduction of the trust factor, especially in e-commerce and online environments, where perceived trust is considered a key factor influencing user acceptance and use of new technologies. The inclusion of trust has enhanced the model’s accuracy in predicting user behavior [37]. Some scholars have also introduced subjective norms and external regulations, Research by Wang et al. [33] found that in the use of AI technology in e-commerce, subjective norms positively influence perceived usefulness and perceived ease of use, and trust has a positive effect on perceived usefulness. Pan et al. [23] studied how TAM-related factors influence two types of usage behaviors on current metaverse platforms. The driving forces for using popular metaverses are perceived usefulness and subjective norms, while the adoption of emerging metaverses is significantly influenced by perceived enjoyment and external regulations. In 2003, Venkatesh et al. [38] proposed the Unified Theory of Acceptance and Use of Technology (UTAUT) model, which adds “facilitating conditions” that influence users’ intentions to accept and use technology as well as actual usage behavior, helping researchers and practitioners better understand the process of technology acceptance.

## 3 Research hypotheses and model construction

### 3.1 Research hypotheses

#### 3.1.1 Antecedent variables

The most prominent issue people face when shopping online is still the lack of physical contact with products and insufficient information about them. Online shopping cannot provide the immediate experience and trial opportunities that physical stores offer. The product images, descriptions, and even videos in online shopping may significantly differ from the actual goods received [39], leading to consumer disappointment and the choice to leave. The ideal solution to this problem is to provide a virtual product experience on consumers’ own shopping devices. AR technology overlays digital information on real-world visual elements, seamlessly integrating virtual products into consumers’ real environments. This not only allows consumers to browse products in entirely new ways but also offers a more personalized and interactive shopping experience, enabling people to have an “immersive” shopping experience without actual contact with the product [14]. With the widespread use of mobile devices such as smartphones and tablets, the application of AR technology in online shopping has become increasingly convenient and popular, changing the way people shop [40]. For instance, virtual try-ons or trials, which are very popular in the fashion and retail industries, allow consumers to virtually try on clothes or shoes, or test various cosmetics on their faces to preview effects before purchasing. They can even try out furniture and decorative items at home, to better understand how these products would look in actual use [11, 41]. AR technology enables interactive product displays, allowing consumers to view 3D models of products through AR apps on their smartphones or tablets, understand products from different angles, zoom in on details, and even observe different configurations and colors of the product [42]. Moreover, AR can create an exciting, enjoyable, and fun atmosphere, providing users with a gamified shopping experience. For example, customers can participate in virtual treasure hunts, searching for specific virtual items in the store to receive discounts or rewards [43]. With these innovative features, AR enhances consumer engagement in online shopping. The AR experience significantly impacts perceived ease of use, leading us to propose the hypothesis:

H1: Sensory experience has a significant impact on perceived ease of use
H2: Emotional experience has a significant impact on perceived ease of use
H3: Cognitive experience has a significant impact on perceived ease of use
H4: Action experience has a significant impact on perceived ease of use
H5: Relational experience has a significant impact on perceived ease of use

Although existing literature has covered various aspects of AR technology, including its applications in fields such as education, healthcare, and entertainment, there has been limited in-depth discussion on its impact in the e-commerce sector, especially in terms of how AR technology influences consumer purchase intentions. Compared to traditional online product displays, AR offers better immersion, novelty, and enjoyment [43], and it has a significantly positive impact on consumers’ online purchase intentions by enhancing user experience [44]. Uhm et al. [10] have further confirmed that augmented reality will improve consumers’ diagnostic perceptions, psychological distance, risk perception, and purchase intentions in e-commerce products, but to varying degrees, with greater impacts on diagnostic perceptions and purchase intentions. Xu et al. [3] identified key AR features in the e-commerce environment and analyzed their effectiveness in helping consumers understand products deeply and creating an engaging atmosphere for customers. Immersive overlays, creative scenarios, and digital twins are important developmental pathways for the e-commerce metaverse [45]. We propose the hypothesis that the AR online shopping experience has a significant impact on perceived usefulness:

H6: Sensory experience has a significant impact on perceived usefulness
H7: Emotional experience has a significant impact on perceived usefulness
H8: Cognitive experience has a significant impact on perceived usefulness
H9: Behavioral experience has a significant impact on perceived usefulness
H10: Relational experience has a significant impact on perceived usefulness

#### 3.1.2 Mediating variables

By integrating AR-based product displays into e-commerce channels, a key goal in the evolution of AR applications in e-commerce is to define and create platforms that merge the physical world of reality with the virtual world of products or services, forming an augmented reality environment. This allows users to overlay and interact with virtual objects within their real-life surroundings, obtain relevant information, engage in creating personalized products, and enhance the shopping experience [13]. Therefore, the characteristics of AR online shopping are reflected in three aspects: vividness, interactivity, and immersion. In the context of e-commerce, vividness is often interpreted as the quality of product presentation [46]. Wang [47] studied the impact of information-oriented and entertainment-oriented smart shopping experiences on consumer purchase intentions. AR technology integrates sensory virtual digital content such as sound, video, graphics, and images, projecting holographic three-dimensional images of products into the surrounding real-world environment in a vivid and novel way [48, 49]. It displays multi-dimensional elements of products, delivering higher quality visual, auditory, and tactile stimuli to media users. This enhances the perceived information quality, expands the number of sensory dimensions a user can experience, and allows users to perceive and interact with virtual elements in a more realistic and three-dimensional manner [50]. Consequently, users can psychologically pre-experience product experiences in future consumption environments, assess the suitability of the products, enhance confidence in their purchasing decisions, and form more enduring memories of the information [45]. Therefore, the following hypotheses are proposed:

H11: Perceived ease of use has a significant impact on purchase intentions
H12: Perceived usefulness has a significant impact on purchase intentions

### 3.2 Model construction

**Fig 1:**
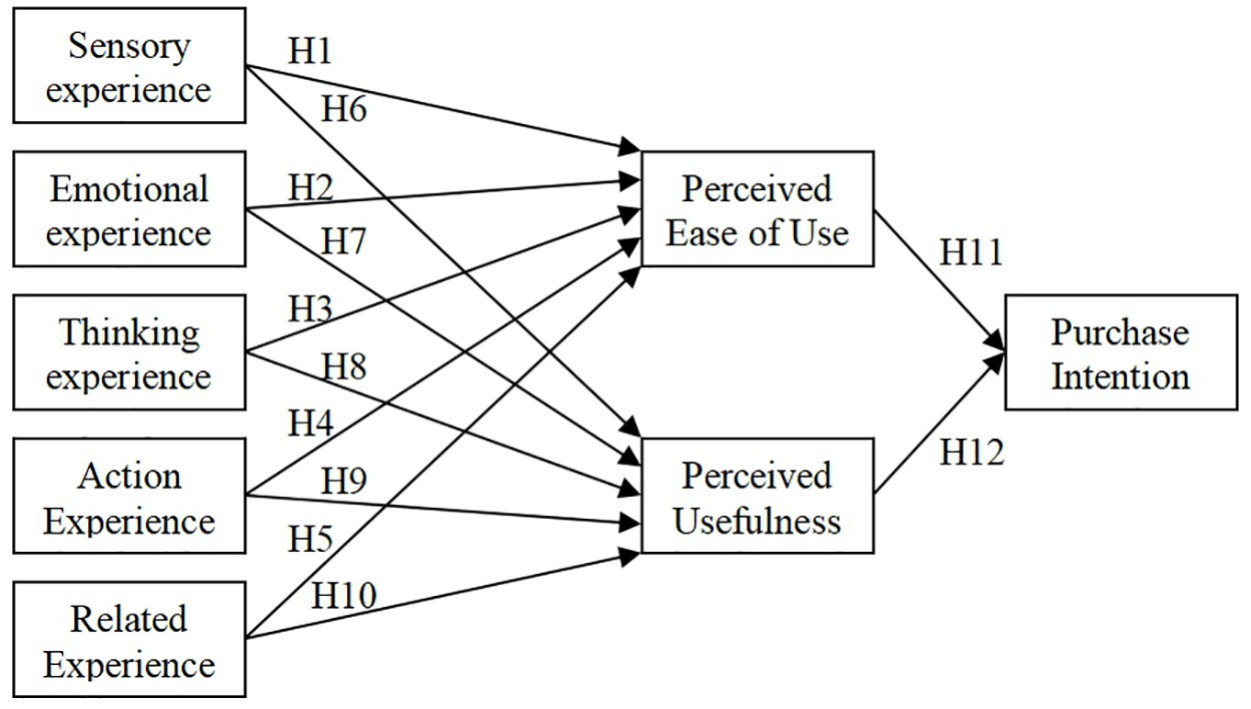
Theoretical model of the impact of AR online shopping experience on customer purchase intention.

Combining the Technology Acceptance Model (TAM), the AR shopping experience incorporates sensory experience, emotional experience, cognitive experience, behavioral experience, and relational experience as antecedent variables. Perceived ease of use and perceived usefulness are treated as mediating variables, and purchase intention as the dependent variable. We construct a theoretical model on the impact of AR online shopping experience on customer purchase intentions, as shown in Fig 1.

### 3.3 Variable measurement

Based on a thorough consideration of related research and practical developments in AR online shopping, a model has been developed to examine the impact of the AR shopping experience on customer purchase intentions. This model consists of eight latent variables (sensory experience, emotional experience, cognitive experience, behavioral experience, relational experience, perceived ease of use, perceived usefulness, and purchase intention) and 30 measurement variables, as seen in Table 3. Each item is measured using a 5-point Likert scale, where 1, 2, 3, 4, and 5 represent “strongly disagree,” “disagree,” “neutral,” “agree,” and “strongly agree,” respectively, allowing respondents to make effective perceptual judgments. The development of the items referred to established scales used in expert and scholarly research and was adjusted according to the characteristics of AR e-commerce shopping, ensuring the accuracy and reliability of the scales.

**Table 3:** Correlated variables and measurements.

SE (Sensory experience)	Sensory experience	SE1	AR online shopping is impressive
		SE2	AR online shopping is rich and entertaining
		SE3	AR product trials have outstanding effects
		SE4	AR online shopping is more realistic
EE (Emotional experience)	Emotional experience	EE1	AR experiences are enjoyable
		EE2	AR online shopping is novel and unique
		EE3	Using AR for products is very interesting
		EE4	AR technology enhances browsing interest
TE (Thinking experience)	Thinking experience	TE1	AR allows for a more extensive and comprehensive understanding of product information
		TE2	AR shopping provides a more intuitive display of outfit effects
		TE3	AR shopping makes it easier to imagine the scenario after purchase
		TE4	AR shopping has changed my perception of traditional e-commerce shopping
AE (Action Experience)	Action Experience	AE1	The application of AR technology brings greater convenience to online shopping
		AE2	AR online shopping can solve more try-on and matching issues
		AE3	AR online shopping changes traditional flat webpage shopping habits
		AE4	Prefer browsing products with AR features
RE (Related Experience)	Related Experience	RE1	AR online shopping enhances interaction with products
		RE2	AR shopping facilitates interaction with backgrounds and matching
		RE3	AR online shopping has certain guided shopping functions
PEOU (Perceived ease of use)	Perceived ease of use	PEOU1	AR online shopping can precisely find products that suit one’s needs
		PEOU2	AR online shopping can enhance the efficiency of e-commerce purchases
		PEOU3	Using AR technology for online shopping is very simple
		PEOU4	AR online shopping can reduce returns due to receiving unsuitable products
PU (Perceived usefulness)	Perceived usefulness	PU1	AR online shopping is very helpful for product selection
		PU2	AR online shopping allows for a preview of the usage effects in advance
		PU3	AR shopping provides additional informational value
		PU4	AR online shopping offers strong references for product matching
PI (Purchase intention)	Purchase intention	PI1	AR technology can stimulate the desire to purchase products
		PI2	More likely to buy products that offer an AR experience
		PI3	Will continue to choose products that offer an AR experience

## 4 Empirical study

### 4.1 Reliability and validity analysis

Based on the measurement items for the relevant variables, a survey questionnaire was developed. The questionnaire focuses on the experiences and evaluations of consumers who have shopped using AR online. The survey was conducted online, targeting AR online shopping consumers, and 279 questionnaires were collected. After discarding invalid questionnaires, 202 valid responses were retained. The standardized Cronbach’s alpha coefficients of the samples are all greater than 0.8, indicating a high level of reliability for the entire survey questionnaire. This suggests that the survey questionnaire is both reliable and stable. Therefore, it is necessary to maintain the measurement items for sensory experience, emotional experience, cognitive experience, behavioral experience, relational experience, perceived ease of use, perceived usefulness, and purchase intention. The Kaiser-Meyer-Olkin (KMO) test statistic is primarily used to compare the simple correlations and partial correlations among variables. When the sum of squares of all simple correlations among variables is significantly greater than the sum of squares of partial correlations, the KMO value approaches 1. The closer the KMO value is to 1, the stronger the correlation among the variables, and the more suitable they are for factor analysis. The KMO values for all variables are not less than 0.7, indicating that factor analysis can be conducted. The Average Variance Extracted (AVE) can test the internal consistency within structural variables. When the AVE value is greater than 0.50, it indicates that the latent variable has good measurement validity. The AVE values for all variables in the table are greater than 0.7, indicating that the validity of the survey questionnaire meets the requirements, as shown in Table 4.

**Table 4:** Reliability and validity analysis of the sample.

Latent Variables	Manifest Variables	Factor Loadings	cronbach’s a	KMO	AVE
Sensory experience	SE1	0.880	0.895	0.848	0.765
	SE2	0.877			
	SE3	0.872			
	SE4	0.870			
Emotional experience	EE1	0.882	0.892	0.845	0.756
	EE2	0.851			
	EE3	0.866			
	EE4	0.878			
Thinking experience	TE1	0.802	0.882	0.832	0.743
	TE2	0.893			
	TE3	0.882			
	TE4	0.868			
Action experience	AE1	0.831	0.872	0.831	0.741
	AE2	0.896			
	AE3	0.851			
	AE4	0.864			
Related experience	RE1	0.901	0.876	0.739	0.803
	RE2	0.880			
	RE3	0.907			
Perceived ease of use	PEOU1	0.885	0.906	0.839	0.781
	PEOU2	0.872			
	PEOU3	0.894			
	PEOU4	0.884			
Perceived usefulness	PU1	0.857	0.903	0.837	0.776
	PU2	0.901			
	PU3	0.889			
	PU4	0.877			
Purchase intention	PI1	0.894	0.876	0.740	0.799
	PI2	0.904			
	PI3	0.884			

The square roots of the AVE for each variable are greater than their correlation coefficients with other variables in the same column, indicating that the measurement scale has good discriminant validity, as shown in Table 5.

**Table 5:** Discriminant validity analysis of the sample.

	**RE**	**AE**	**TE**	**EE**	**SE**	**PU**	**PEOU**	**PI**
RE	**0.896**							
AE	0.000	**0.861**						
TE	0.000	0.000	**0.862**					
EE	0.000	0.000	0.000	**0.869**				
SE	0.000	0.000	0.000	0.000	**0.875**			
PU	0.133	0.234	0.082	0.126	0.093	**0.881**		
PEOU	0.127	0.628	0.121	0.060	0.248	0.253	**0.884**	
PI	0.144	0.224	0.090	0.130	0.089	0.168	0.238	**0.894**

Note: The diagonal values represent the square roots of the AVE of the corresponding latent variables.

### 4.2 Estimation of structural equation model

**Fig 2:**
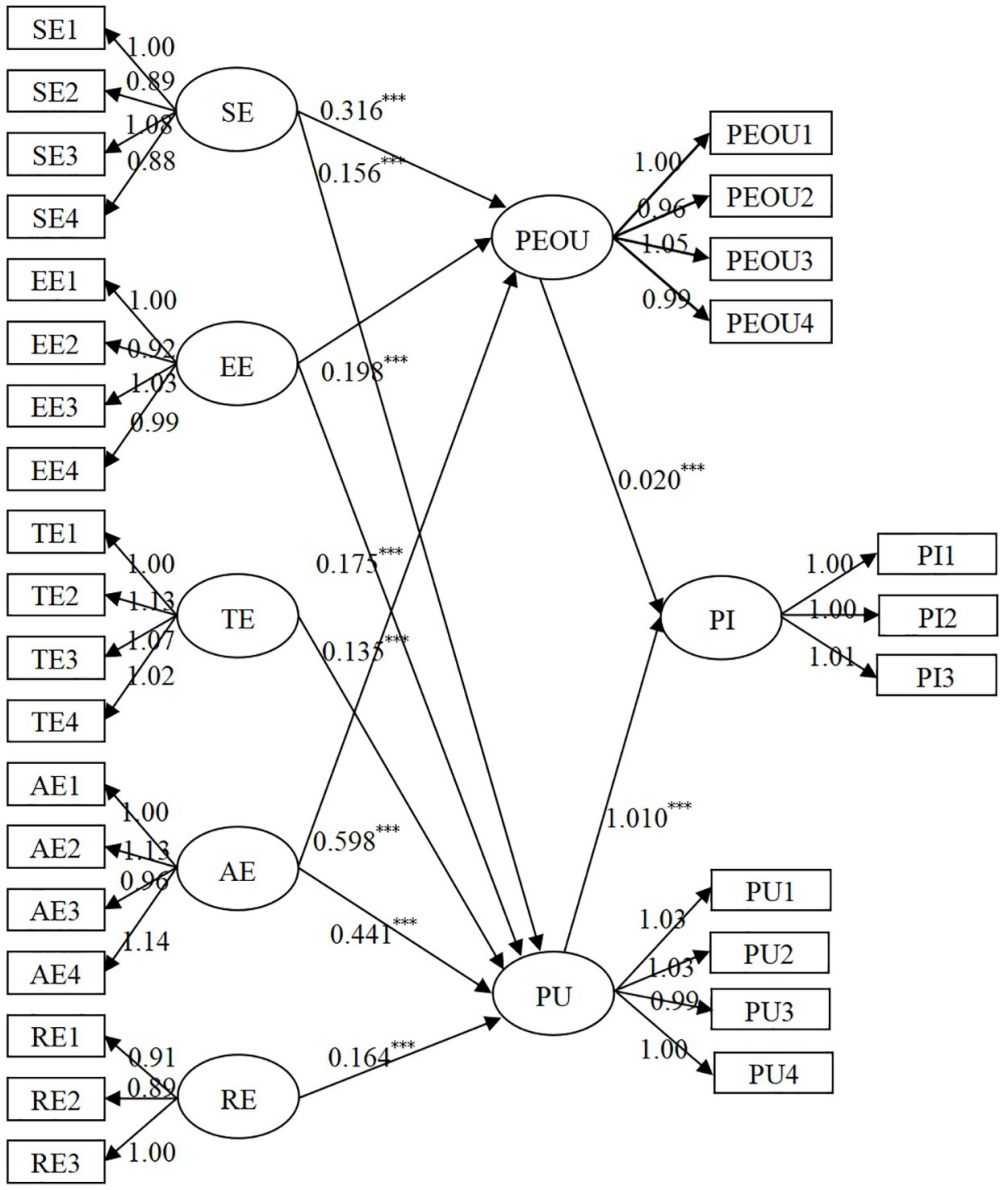
Revised structural equation model and path coefficients. Note: *** indicates that the significance (sig) value is less than 0.001.

Using AMOS 22 software to fit the structural equation model, the initial structural equation model yielded T-values of -4.779 for “TE→PEOU” and -4.526 for “RE→PEOU,” which do not meet the standard of T-values > 1.96. After removing the two non-significant paths “TE→PEOU” and “RE→PEOU,” the model was refitted, resulting in the revised structural equation model and path coefficients as shown in Fig 2.

In the revised model’s path coefficient test results, all path T-values exceeded the minimum standard of 1.96, and all p-values were significant at the 0.001 level. Overall, the path coefficients in the revised model are quite significant. From the perspective of various fit indices, the structural equation model has a χ2/df value of 4.601, which is less than 10; GFI value of 0.810, close to 1; AGFI value of 0.742, close to 1; RMSEA value of 0.034, less than 0.05; and NFI, CFI, and IFI values are 0.708, 0.754, and 0.756, respectively, all close to 1, as shown in Table 6. These results indicate that the model fits well and has good adaptability, and the model should be accepted.

**Table 6:** Fit analysis of the revised model.

Name of Fit Index		Judgment	Model Fit Value	Model Fit Status
Absolute Fit Index	*χ* ^*2*^ */df*	<10	4.601	Conforms
	GFI	Close to 1	0.810	Conforms
	AGFI	Close to 1	0.742	Conforms
	RMSEA	<0.05	0.034	Conforms
Relative Fit Index	NFI	Close to 1	0.708	Conforms
	CFI	Close to 1	0.754	Conforms
	IFI	Close to 1	0.756	Conforms

Using the Bootstrap method to test for mediating effects, the sample was bootstrapped 5000 times with replacement at a 95% confidence level, and the results indicate the presence of mediating effects, as shown in Table 7.

**Table 7:** Test of mediating effects.

Mediating Effect	Total Effect	Path	Path Coefficient	95% Confidence Interval
SE→PI	0.164	SE→PEOU→PI	(SE→PEOU)*(PEOU→PI) = 0.316*0.020 = 0.006	[0.016,0.213]
		SE→PU→PI	(SE→PU)*(PU→PI) = 0.156*1.010 = 0.158	
EE→PI	0.181	EE→PEOU→PI	(EE→PEOU)*(PEOU→PI) = 0.198*0.020 = 0.004	[0.034,0.387]
		EE→PU→PI	(EE→PU)*(PU→PI) = 0.175*1.010 = 0.177	
TE→PI	0.136	TE→PU→PI	(TE→PU)*(PU→PI) = 0.135*1.010 = 0.136	[0.004,0.288]
AE→PI	0.563	AE→PEOU→PI	(AE→PEOU)*(PEOU→PI) = 0.589*0.020 = 0.118	[0.366,0.741]
		AE→PU→PI	(AE→PU)*(PU→PI) = 0.441*1.010 = 0.445	
RE→PI	0.611	RE→PU→PI	(RE→PU)*(PU→PI) = 0.164*1.010 = 0.166	[0.389,0.776]

### 4.3 Hypothesis testing

The study demonstrates significant effects of sensory experience (SE) on perceived ease of use (PEOU), emotional experience (EE) on PEOU, behavioral experience (AE) on PEOU, sensory experience on perceived usefulness (PU), emotional experience on PU, cognitive experience (TE) on PU, behavioral experience on PU, and relational experience (RE) on PU. Additionally, PEOU on purchase intention (PI) and PU on PI are significantly impacted. However, the hypotheses that relational experience significantly affects PEOU and that cognitive experience significantly affects PEOU are not supported, as shown in Table 8.

**Table 8:** Summary of hypothesis testing.

Number	Research Hypothesis	Verification Result	Number	Research Hypothesis	Verification Result
H1	SE→PEOU has a significant impact	Supported	H7	EE→PU has a significant impact	Supported
H2	EE→PEOU has a significant impact	Supported	H8	TE→PU has a significant impact	Supported
H3	TE→PEOU has a significant impact	Not supported	H9	AE→PU has a significant impact	Supported
H4	AE→PEOU has a significant impact	Supported	H10	RE→PU has a significant impact	Supported
H5	RE→PEOU has a significant impact	Not supported	H11	PEOU→PI has a significant impact	Supported
H6	SE→PU has a significant impact	Supported	H12	PU→PI has a significant impact	Supported

**Fig 3:**
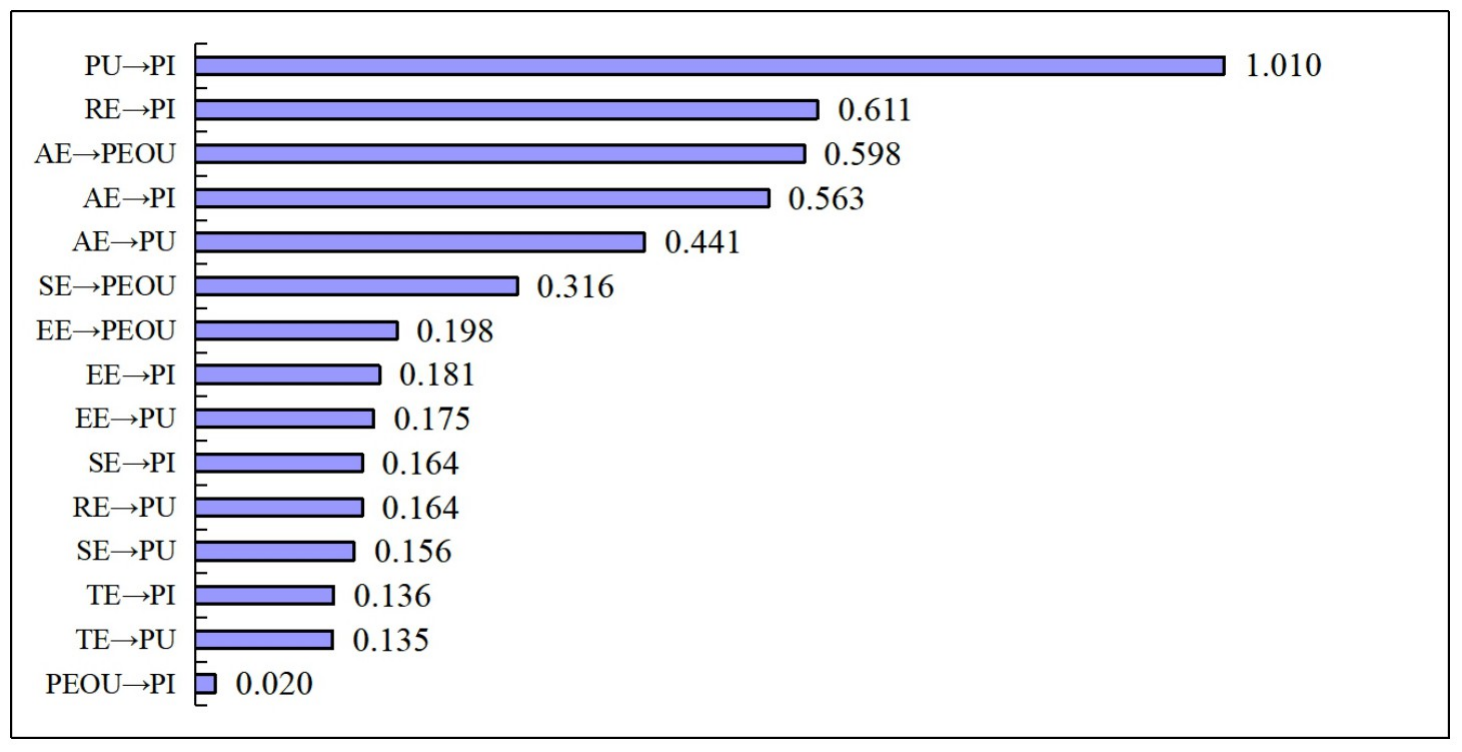
The impact of AR online shopping experience on customer purchase intentions.

Among all the effects, the impact of PU on PI is the greatest, with a coefficient of 1.010; followed by the impact of RE on PI, with a coefficient of 0.611; the third highest is the impact of AE on PEOU, with a coefficient of 0.598; the fourth is the impact of AE on PI, with a coefficient of 0.563; the smallest impact is from PEOU on PI, with a coefficient of 0.020, as detailed in Fig 3.

## 5 Discussion

### 5.1 Conclusions

This study extends the Technology Acceptance Model (TAM) by incorporating five types of AR online shopping experiences (sensory experience, emotional experience, cognitive experience, action experience, and relational experience) as antecedent variables, with perceived ease of use (PEOU) and perceived usefulness (PU) as mediating variables. A structural equation model was constructed and empirically tested to explore the impact mechanisms of AR online shopping experiences on customer purchase intention. The main findings are as follows:

1. Positive Impact of Sensory Experience: The vividness, interactivity, and immersive sensory experience of AR enhance the perceived ease of use and perceived usefulness of online shopping for consumers.
2. Role of Emotional Experience: The positive emotions triggered by AR improve consumers’ perceptions of the usability and usefulness of online platforms, increasing their shopping pleasure and utility. This supports the findings of studies [3, 8, 41].
3. Impact of Cognitive Experience: Cognitive experience significantly influences perceived usefulness but does not affect perceived ease of use. This indicates that the comprehensive and detailed understanding of product information, the vivid presentation of matching effects, the display of post-purchase usage scenarios, interaction with products, matching of related products and scenes, and enhancement of guidance functions provided by AR are very valuable and practical for providing information and aiding decision-making. However, in actual operation, consumers may still find using AR technology somewhat complex, and thus it does not significantly simplify the shopping process or improve shopping efficiency.
4. Contribution of Action and Relational Experiences: Action and relational experiences enhance shopping experiences and social interactions, leading to stronger purchase intentions among consumers. Although relational experience does not significantly affect perceived ease of use, it enhances consumers’ sense of social recognition, consistent with the conclusion in the literature [11] that AR stimulates purchase intentions in shopping environments.
5. Mediating Role of Perceived Ease of Use and Perceived Usefulness: Both perceived ease of use and perceived usefulness significantly influence purchase intention, with the impact of perceived usefulness being the greatest. This indicates that although ease of use contributes to an improved shopping experience, it does not significantly drive purchase decisions unless it provides substantial practical utility. This finding aligns with the conclusion in the literature [5] about the mediating role of perceived value in AR usage motivation and purchase intention.

In summary, this study expands the application scope of the Technology Acceptance Model (TAM), providing new insights into how different types of AR experiences influence consumer behavior. It reveals the multiple impact mechanisms of AR online shopping experiences on customer purchase intention, enriching the theory of consumer behavior in Metaverse e-commerce.

### 5.2 Recommendations

#### 5.2.1 Enhancing scenario construction to empower AR online shopping experience

**Fig 4:**
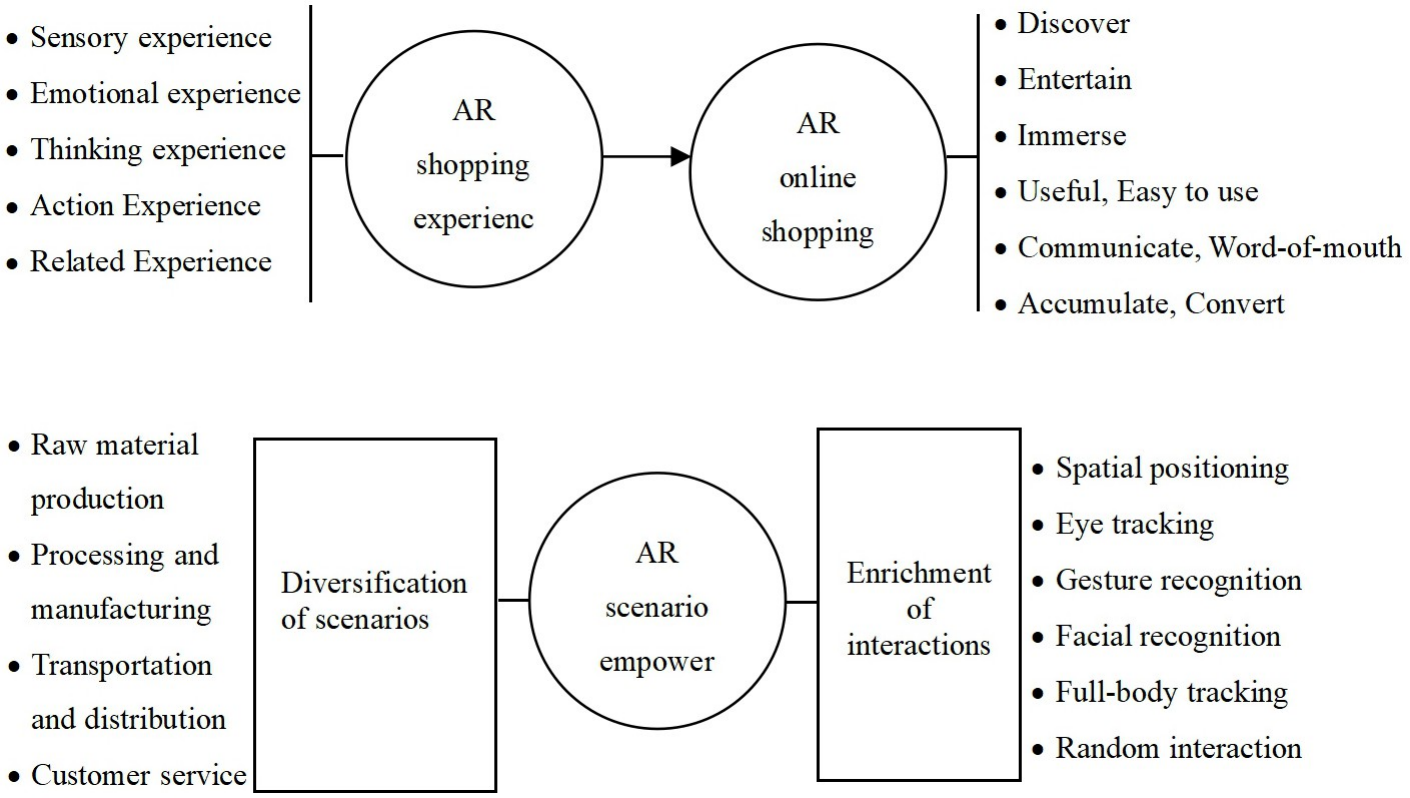
AR shopping scenario empowering experience and consumer online shopping.

The creation of AR scenarios is a crucial step in enhancing the online shopping experience and boosting purchasing intentions. Empowering AR scenarios includes two major aspects. First is the diversification of scenario construction. Currently, AR online shopping scenarios are mainly focused on product demonstrations. Further development needs to create more diversified scenarios, including the integration of AR technology in the production of raw materials, product manufacturing, warehousing and transportation, customer service, and live commerce, allowing consumers to have a more direct and enhanced experience of the entire supply chain. Second is the enrichment of interactive development. Current interactions focus on gesture recognition, but there is a need to further develop technologies such as spatial positioning, eye-tracking, facial recognition, full-body tracking, and random interaction in AR shopping to more accurately determine the shopping space, analyze consumer emotions, display full-body effects and overall environmental effects, and enhance the level of interaction. AR scenario empowerment can simultaneously enhance the five major experiences of AR shopping and positively impact consumers’ online shopping intentions in terms of product discovery, leisure and entertainment, enhanced immersion, improved usefulness and ease of use, promotion of communication, development of word-of-mouth, strengthening brand consolidation, and facilitating commercial conversion, as shown in Fig 4.

#### 5.2.2 Enhancing sensory experience in AR online shopping

Utilize high-definition images and advanced rendering technologies to create realistic 3D models, enhancing the detail and authenticity of products displayed in an AR environment. Incorporate unique visual effects, such as dynamic lighting and shadows or adding interactive elements, to make the user experience more engaging and memorable. Develop a variety of AR application scenarios, allowing users to experience the effects of products in their own environment. Guide users through AR games or interactive tutorials to learn about products, providing an educational and entertaining shopping experience. Design personalized shopping paths that allow users to explore actively within the AR environment based on their interests and shopping habits. Offer unique AR product trial experiences that include multisensory elements, such as simulating the texture and color changes of products, and even providing olfactory and gustatory stimuli, enabling users to feel the products more genuinely and ensuring that the AR trial features align closely with the actual quality and characteristics of the products. Provide highly customized experiences, allowing users to adjust the product’s color, size, or design to suit their personal preferences.

#### 5.2.3 Enhancing the emotional experience in AR online shopping

Design beautiful, vivid, and attractive AR interfaces to create compelling immersive effects. Analyze user preferences scientifically based on their shopping history data and recommend customized AR product displays. Provide diverse AR display options, such as 3D views and 360-degree rotations, allowing users to explore products from multiple angles and details. Integrate emotional elements into the products, such as using AR to display the product’s story or origins and employing narrative techniques to present products, allowing users to enjoy the storyline while exploring the product, enhancing the emotional connection between users and products. Merge metaverse and virtual reality technologies to create a new shopping environment that transcends traditional online shopping, enabling users to experience products in a novel and fully immersive way. Offer lively and interactive experiences, such as incorporating gamified elements, where users can earn discounts or points by completing small tasks within the AR experience; allow users to customize or experiment with products using AR technology.

#### 5.2.4 Enhancing the cognitive experience in AR online shopping

Utilize AR technology to provide detailed and comprehensive product information, including 3D models that show every angle and detail of the product; incorporate enhanced description features that automatically display related product specifications, materials, or usage methods when users view specific parts. Offer additional information related to the product, such as customer reviews, production background, and usage scenarios, enabling users to fully understand the product information. Visually demonstrate pairing effects, using AR technology for virtual try-ons or home setups, allowing users to see the product pairing effects intuitively. Provide diverse pairing options and suggestions to help users explore different styles or design proposals. Allow users to freely mix and match different products in a virtual environment, increasing space for experimentation and innovation, and better imagine scenarios post-purchase. Create realistic post-purchase usage scenario simulations, such as allowing users to see the product’s effect in their own home or anticipated usage environment. Integrate emotional elements, such as simulating users’ feelings or life improvement effects after using the product, enhancing emotional resonance. Transform perceptions of traditional e-commerce, emphasizing the unique value provided by AR shopping, such as higher interactivity and more accurate product experiences. Educate and guide users to understand the advantages of AR shopping, such as accuracy, convenience, and personalized experiences. Analyze new insights and feelings gained by users through AR shopping, and how this influences their shopping decision process.

#### 5.2.5 Optimizing the behavioral experience in AR online shopping

Enhance shopping convenience by developing intuitive and user-friendly AR application interfaces, ensuring that users of all ages and technical levels can easily utilize them. Simplify the shopping process through AR technology, such as implementing one-click shopping, allowing users to directly select and purchase products within the AR experience. Provide efficient product search and filtering tools, enabling users to quickly find the AR experience products they need. Offer dynamic pairing suggestions to help users choose the right product combinations based on their personal style and occasion needs. Enable users to experience the effects of different product combinations at home through virtual try-on and pairing features, reducing the hassles of purchase errors and returns. Shift traditional shopping habits by emphasizing the advantages of AR shopping over traditional flat webpage shopping, such as more realistic product previews and higher interactivity. Educate users on how to effectively use AR technology for shopping, helping them adapt to this new mode of shopping through case demonstrations or tutorials. Encourage merchants to incorporate AR experiences into product displays, enhancing users’ affinity for AR-capable products by providing richer and more in-depth product information, and attracting users with more vivid and immersive shopping experiences. Collect user feedback and continuously improve the AR shopping experience to ensure it meets user needs and exceeds expectations.

#### 5.2.6 Enhancing the relational experience in AR online shopping

Utilize AR technology to provide direct interaction with products, such as allowing users to rotate, zoom in, and zoom out on product models via gestures or touch, and even try on or test products. Create an interactive virtual environment, for example, by simulating actual usage scenarios, allowing users to experience products in novel ways. Develop AR tools that enable users to virtually place products in their own environments to assess their adaptability and aesthetic fit. Offer virtual pairing suggestions, such as automatically displaying other items that complement the selected product or suggested pairing methods. Facilitate user interaction with the pairing scenario, such as adjusting the lighting or background in the scene to better display the product effects. Act as a shopping guide by using AR technology to provide personalized shopping suggestions, such as recommending products based on a user’s shopping history and preferences. Integrate chatbots or virtual shopping assistants to provide real-time answers and advice, enhancing the interactivity and helpfulness of the shopping experience.

## 6 Limitations and future directions

The limitations of this study are primarily reflected in the data collection process. The quantitative data used for the structural equation modeling (SEM) analysis were obtained through a cross-sectional survey conducted in China. The sample was concentrated on specific demographic characteristics or geographic regions. The cross-sectional study design only captures data at a single point in time, failing to reveal the long-term impact of AR online shopping experiences on purchase intention, thus limiting the generalizability of the study results. Future research should consider more diverse samples to validate the applicability of the findings across different populations and regions. Additionally, studies should adopt longitudinal designs to track changes in consumer behavior before and after using AR technology, to understand the long-term impact of AR shopping experiences on purchase intention and potential behavior changes. Secondly, this study primarily focused on the impact of AR technology on online shopping experiences and customer purchase intention. Future research could further explore the impact of combining AR and artificial intelligence technologies on e-commerce customer online shopping, as well as investigate the patterns of consumer behavior in the metaverse e-commerce environment.

## Supporting information

S1 Data Raw data and the means, standard deviations, variances, minimum, and maximum values of the raw data. (XLSX)
